# Diagnostic Yield and Safety of Pulmonologist-Performed Ultrasound-Guided Transthoracic Core Biopsy: A Seven-Year Cohort Study

**DOI:** 10.3390/diagnostics16121913

**Published:** 2026-06-19

**Authors:** Ruxandra Mioara Râjnoveanu, Adriana Părău, Gabriel Flaviu Brișan, Mădălina Valeanu, Jenica Maria Șimon, Doina Adina Todea, Milena Adina Man, Corina Eugenia Budin, Vlad Alexandru Harnuț, Bogdan Fetica, Armand Gabriel Râjnoveanu

**Affiliations:** 1 Palliative Medicine Department, Iuliu Hatieganu University of Medicine and Pharmacy, 400371 Cluj-Napoca, Romania; ruxandra.rajnoveanu@umfcluj.ro; 2 Leon Daniello Pneumology Hospital, 400371 Cluj-Napoca, Romania; adrianaparau@yahoo.com; 3 Pneumology Department, Iuliu Hatieganu University of Medicine and Pharmacy, 400371 Cluj-Napoca, Romania; jenica.simon@yahoo.com (J.M.Ș.); dtodea@umfcluj.ro (D.A.T.); manmilena50@yahoo.com (M.A.M.); harnutvladalexandru@yahoo.com (V.A.H.); 4 Department of Medical Informatics and Biostatistics, Iuliu Hatieganu University of Medicine and Pharmacy, Louis Pasteur Street, no. 6, 400349 Cluj-Napoca, Romania; mvaleanu@umfcluj.ro; 5 Pathophysiology Department, George Emil Palade University of Medicine, Pharmacy, Science and Technology of Targu Mures, 540142 Targu Mures, Romania; corina.budin@umfst.ro; 6 Pathology Department, Iuliu Hatieganu University of Medicine and Pharmacy, 400337 Cluj-Napoca, Romania; feticab@yahoo.com; 7 Occupational Medicine Department, Iuliu Hatieganu University of Medicine and Pharmacy, 400349 Cluj-Napoca, Romania; armand.rajnoveanu@umfcluj.ro

**Keywords:** lung cancer, thoracic ultrasound, transthoracic core biopsy, pulmonologist-performed, Clavien–Dindo classification, diagnostic yield, learning curve

## Abstract

**Background/Objectives**: Given rising lung cancer incidence and limited data on pulmonologist-performed ultrasound-guided transthoracic core biopsy (US-TTCB), in this study, we evaluated diagnostic yield and safety for pleural or pulmonary lung masses, using Clavien–Dindo classification to standardize complication reporting. **Methods**: We retrospectively reviewed single-center pulmonologist-performed US-TTCB using a MEDONE biopsy gun with a 16 G/18 G Tru-Cut needle between January 2019 and December 2025. The primary endpoints were diagnostic yield, defined as specific malignant or benign histology, and complication rate. Non-diagnostic results were assessed using available clinical/imaging follow-up. Univariate analyses screened candidate correlates, and a prespecified computer tomography (CT)-completed subanalysis (*n* = 67) used multivariable logistic regression and receiver operating characteristic (ROC) analysis to assess CT lesion size discrimination. **Results**: Diagnostic yield was 84.2% (202/240); complications occurred in 12.1% (29/240), including one Clavien–Dindo Grade III event (0.4%). In the CT-completed subset (*n* = 67), diagnostic yield was independently associated with CT lesion size (aOR 1.03/mm, 95% CI 1.00–1.05; *p* = 0.022) and Chronic Obstructive Pulmonary Disease (COPD) (aOR 2.30, 95% CI 1.06–4.96; *p* = 0.034); CT lesion size showed an area under the curve (AUC) of 0.717 for predicting yield. Diagnostic yield remained stable over time (84.2% in first vs. second half; *p* = 1.00), with no association between case order and yield (OR 0.999; *p* = 0.64). **Conclusions**: US-TTCB of pleural/pulmonary masses achieved a high diagnostic yield with minimal major complications. Large CT dimension and COPD were associated with higher diagnostic success, and CT size provided fair discrimination for predicting yield; findings should be interpreted in the context of the retrospective single-center design and the restricted CT-completed subset.

## 1. Introduction

In modern respiratory medicine, peripheral pulmonary lesions (PPLs) and pleural modifications represent a significant diagnostic challenge. With the availability of high-resolution computer tomography (CT) and the implementation of large-scale screening programs, clinicians are increasingly tasked with providing a feasible biopsy procedure to ensure a proper histopathological diagnosis with minimal risks [1,2].

For decades, CT-guided transthoracic needle biopsy (CT-TTNB) has served as the conventional ‘gold standard’ for sampling pulmonary peripheral lesions. However, the past decade has seen a paradigm shift in conventional methods, with US-TTCB positioning itself as a top choice when it comes to the diagnosis of pleural or peripheral lung lesions [3]. Unlike CT, ultrasound allows continuous, real-time visualization of the needle trajectory, a detrimental part of the biopsy process, allowing the operator to compensate for respiratory motion, a frequent cause of sampling error in static imaging. Furthermore, US-TTCB reduces ‘diagnostic delay’ by bypassing the need for inter-departmental scheduling in radiology suites and facilitating a bedside approach [4,5].

The current state of research highlights a clear advantage of US-TTCB in terms of diagnostic yield and safety. A recent meta-analysis (2023) reported for US-TTNB a pooled sensitivity and specificity of 0.93 and 0.99, respectively; while CT-TTNB remains a high-yield reference procedure, US-TTCB may represent a valuable alternative for pleural-based lesions [3].

In terms of safety, a recent meta-analysis and multicenter trials have consistently demonstrated that US-TTCB carries a lower risk of iatrogenic pneumothorax (<5%) compared to CT-guided methods (15–25%) [6,7,8]. This is primarily due to the ‘sonographic window’ request; only lesions in contact with the pleura can be visualized and therefore no air is placed in the trajectory of the needle, avoiding the risk of air leakage [9].

However, particular attention is given to the management and classification of ‘non-diagnostic’ samples, i.e., those presenting only necrosis, non-specific inflammation, or respiratory mucosa. While the result is seen as a procedural failure requiring immediate re-intervention, others propose a ‘conservative follow-up’ model [10]. There is also ongoing debate regarding the standardization of complication reporting in interventional pulmonology. While the Clavien–Dindo classification is the established language for surgical complications, its application to percutaneous needle biopsies is a relatively recent development aimed at improving the transparency and comparability of safety data across different medical centers [11,12].

In Romania, while interventional pulmonology is an advancing discipline, there remains a paucity of long-term, high-volume data reflecting real-world clinical outcomes. The primary aim of this work is to evaluate the diagnostic performance and safety profile of a freehand US-TTCB technique performed by a single operator over a seven-year period (2019–2025) at a tertiary respiratory/thoracic surgery center. By analyzing a cohort of 240 patients and employing a 12-month follow-up protocol for non-diagnostic results, we demonstrated that US-TTCB is a high-yield, low-risk diagnostic tool.

## 2. Materials and Methods

### 2.1. Study Design and Population

We conducted a retrospective analysis of a prospective maintained procedural database at the ‘Leon-Daniello’ Clinical Hospital of Pneumology in Cluj-Napoca, Romania. The study period included all consecutive adult patients who underwent US-TTCB between January 2019 and December 2025.

The study was approved by the Institutional Review Board of the ‘Leon Daniello’ Pneumology Hospital, Cluj-Napoca (No 16/17.12.2025, date 17 December 2025). All patients provided written informed consent for the US-TTCB as part of routing clinical care. Before the procedure, the performing physician explained the indication, technical steps, expected benefits, and possible complications, and patients were given the opportunity to ask questions before signing the institutional procedural consent form. Because this was a retrospective analysis of routinely collected clinical data, the requirement for specific informed consent for study inclusion was waived by the Institutional Review Board. Patient data were anonymized before analysis to protect confidentiality.

This study follows the Strengthening the Reporting of OBservational studies in Epidemiology (STROBE) guidelines (Supplementary File S1).

### 2.2. Patient Selection and Eligibility

The interventional pulmonologist who initiated the US-TTCB program in 2019 consecutively recorded all patients undergoing the procedure.

Inclusion criteria were:

- Patients aged ≥ 18 years;
- Presence of pulmonary consolidation (tumor-like or pneumonic) with minimal diameter of 20 mm along the needle trajectory (required to accommodate the technical ‘throw’ of the cutting needle);
- Fixed parietal contact (absence of interposition of air during respiration);
- Ultrasound-detectable pleural thickening or pleural tumors.

Exclusion criteria were lesions lacking static parietal contact, suspected vascular etiology (e.g., malformations or aneurysms), lesions < 20 mm, in proximity to vital organs, extensive emphysema in the needle path, unstable angina or recent myocardial infarction, uncorrected coagulopathy, severe respiratory failure, inability to cooperate with breathing instructions, or patient refusal.

### 2.3. Technical Procedure

All procedures were performed by a single pulmonologist with extensive prior experience in bronchology and thoracic and general ultrasonography, including a certificated course and training in interventional ultrasound. A SonoScape S30 ultrasound system (SonoScape Medical Corp., Shenzhen, China) equipped with a convex probe (2–7 MHz) was used. Following CT review and baseline ultrasound, the patient was positioned in such a way as to provide the most direct and comfortable access to the lesion. Before each biopsy, the size and surrounding structures of the target lesion, and the optimal needle depth and path were assessed. Local anesthesia was achieved using 10–30 mL of Lidocaine 1%, not exceeding 1 mg/kg. Biopsies were obtained via a freehand technique without the use of electronic markers or contrast agents; Doppler mode was used to avoid vascular structures, such as vessels in pneumonic/atelectatic lesions or intercostal vessels. The probe was cleaned with iodine solution and then put on a sterile glove to ensure sterilization. One to four tissue samples were usually collected per session. Biopsy was performed, under US guidance, using a MEDONE gun (Medax Srl Unipersonale, San Possidonio, Italy) with a 16 G or 18 G automated disposable Tru-Cut needle, with immediately preservation of samples in 9% formalin until histopathological analysis. Neither coaxial needles nor contrast enhancement was used.

### 2.4. Data Collection and Variables

Access to the hospital electronic medical records was provided through Info World (Hospital Manager Suiteș S.C. Info World S.R.L.), running version FP 17 (build 2.5.331), a ward information system that was used to retrieve demographics, clinical variables, procedural documentation, complications, and follow-up information. Data integrity was ensured by two investigators who validated the database against clinical records and histopathology reports using names and personal identification numbers.

The variables collected were as follows:

- Demographics and Socioeconomics: Age, sex, residence (urban/rural), and occupation.
- Clinical History: Smoking status, pack-year index, and presence of COPD or emphysema.
- Lesion Characteristics: Anatomical location, size, and sonographic/computer tomography appearance. We also recorded associated findings such as pleural effusion, lymphadenopathy, and suspected metastasis.
- Procedural Details: Patient position, needle gauge, number of biopsy passes, and procedure data.
- Outcomes: Final histological diagnosis, total length of stay (days), and post-intervention complications.

### 2.5. Complications and Safety Assessment

Complications were recorded and graded according to the Clavien–Dindo classification system (Appendix A Table A1). To maintain transparency, in instances where the medical documentation provided an incomplete description of the clinical response to a complication, we reviewed medication records and discharge summaries to assign the correct complication grade.

### 2.6. Diagnostic Performance and Follow-Up Protocol

The primary outcome was diagnostic yield, defined as the percentage of biopsies providing a definitive malignant or specific benign histopathological diagnosis. Non-diagnostic results included samples showing only necrosis, non-specific inflammation, insufficient tissue, or normal respiratory mucosa.

Secondary Outcome—False Negative Assessment.

For patients with non-diagnostic initial results, we retrospectively reviewed the Info World system to identify subsequent diagnostic procedures and clinical outcomes over a minimum 6-month period following the index biopsy (up to December 2025).

Final diagnoses were established through repeated biopsies (surgical or percutaneous), surgical resection with pathology, clinical/radiological stability or resolution (considered benign), and clinical progression or new pathological confirmation (false negative).

Cases with documented lesion stability or resolution on follow-up imaging (minimum 6 months) without intervening diagnosis were considered true negatives for malignancy. Cases with subsequent confirmed malignancy or specific benign diagnosis via alternative methods were classified as false negatives.

Of 52 initially non-diagnostic patients, 25 (48%) had insufficient follow-up data in the Info World system (transferred care, lost to follow-up, follow-up imaging to assess stability <6 months, or incomplete records) and were retained as ‘non-diagnostic’ in the primary analysis, accepting the initial report and using a conservative intention-to-diagnostic approach.

### 2.7. Statistical Analysis

Data were analyzed using IMB SPSS Statistics for Windows, Version 25.0 (IBM Corp., Armonk, NY, USA) and R software, version 4.4.1. Descriptive statistics summarized patient demographics, lesion characteristics, histological diagnoses, and procedure-related technical details. Categorical variables are presented as frequencies and percentages, while continuous variables are expressed as mean ± standard deviation (SD) or median with interquartile range (IQR), depending on the distribution. The learning curve was assessed by comparing diagnostic yield over time, including first versus second half of the cohort and quartiles of procedural sequence, and by modeling case order as a continuous predictor of diagnostic yield using logistic regression.

Independent predictors of diagnostic yield and complication rate were evaluated using univariate analysis. A multivariable logistic regression model was then fitted with diagnostic yield (diagnostic vs. non-diagnostic biopsy) as the dependent variable; CT lesion size, COPD, and pleural effusion were included as covariates on the basis of clinical relevance and univariate findings. Adjusted odds ratios (ORs) with 95% confidence intervals (CIs) are reported. Because CT lesion size was available only in a subset of patients, the final model was based on complete-case analysis and included 67 patients. ROC analysis was used to assess the discriminative performance of CT lesion size for diagnostic yield.

## 3. Results

**Figure 1:**
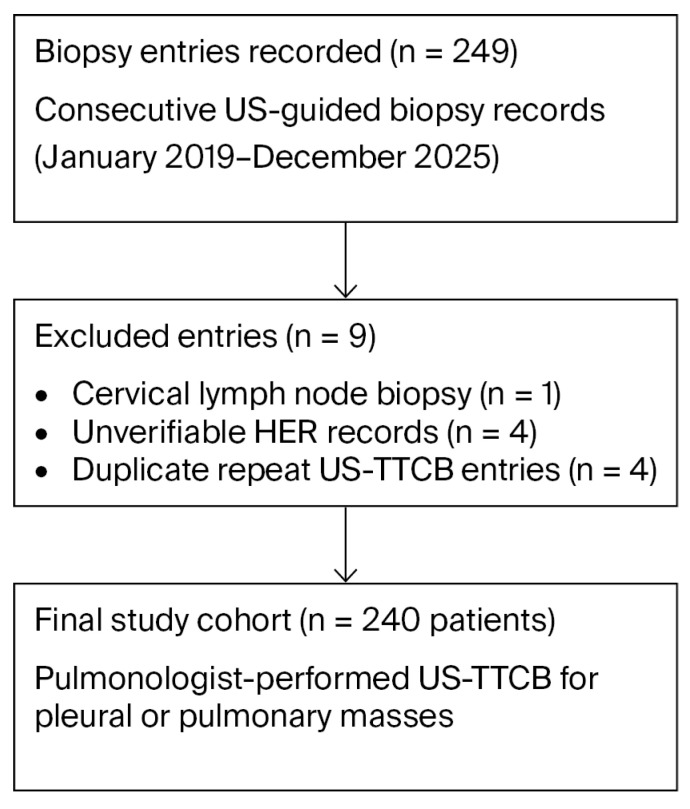
Study flow diagram for screening of biopsy entries.

Initially, 249 biopsy entries were recorded. We excluded one patient with a cervical lymph node biopsy, four patients with unverifiable records in the Info World system, and four duplicated entries from multiple US-TTCB. The patient selection process is summarized in Figure 1.

Baseline demographic and clinical characteristics (Table 1), lesion characteristics (Table 2), procedural and technical characteristics (Table 3), and study primary endpoints (Table 4) are summarized. Because not all variables were available for all patients, the number of observations varied across analyses. Data are presented as median (IQR) or *n*/*N* (%), as appropriate. The final cohort consisted of 240 patients, with a predominance of men (70.0%) and a median age of 67.0 years (IQR 60.8–74.0).

Lesions were predominantly located in the lower lobes, with a median ultrasound size of 40 mm (IQR 30–60); CT-based size measurements were available in a subset (*n* = 76), with a median CT size of 60 mm (IQR 43.5–87.5). Most biopsies yielded malignant histology (75.4%).

The main study findings were a diagnostic yield of 84.2% and a low major complication rate of 0.4%, while recorded complications occurred in 12.1% of cases.

Histology data were available for all 240 patients along with ultrasound lesion size (Table 5). Adenocarcinoma was the most frequent biopsy diagnosis (35.4%), followed by squamous cell carcinoma (14.4%) and inflammatory lesions (12.5%).

Percentages are calculated out of 240 cases and may not total 100.0% because of rounding.

In the full cohort, 202 (84.2%) biopsies were diagnostic and 38 (15.8%) were evaluated as non-diagnostic. In the exploratory univariate analysis (Table 6), diagnostic yield was higher for larger lesions, both on CT (69.6 ± 35.4 vs. 44.0 ± 25.1 mm; *p* = 0.0098) and on US (45.0 [30.0–60.0] vs. 37.5 [30.0–49.0] mm; *p* = 0.0402). Patient position was also associated (*p* = 0.045), with non-diagnostic biopsies more frequently performed in ventral decubitus (47.4% vs. 24.3%). COPD and pleural effusion presented borderline associations (*p* = 0.068 and *p* = 0.089), while all other variables were not significant (*p* > 0.055).

Variables with clinical relevance and/or *p* < 0.10 were evaluated in a multivariable logistic regression (Table 7) using the complete-case CT subset (*n* = 67). Patient position was not retained because of sparse category distribution and unstable estimates, particularly for lateral decubitus positioning.

**Figure 2:**
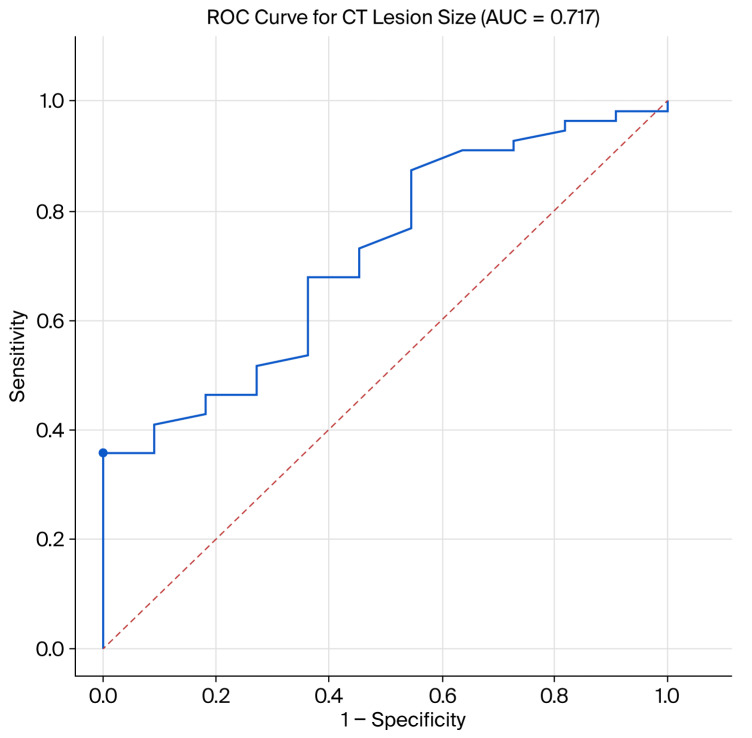
ROC curve evaluating the predictive performance of lesion size on CT for diagnostic yield, including 56 diagnostic cases and 11 non-diagnostic ones.

ROC analysis (AUC = 0.717) in Figure 2. showed fair discriminatory ability of lesion size for diagnostic yield.

### 3.1. Complications Evaluation

Total complications: 29 cases (12.1%).

- Grade I: 24 (84.8%).
- Grade II: 4 (14.8%).
- Grade III: 1 (0.4%).

The procedure was assessed as being safe because the majority of complications were classified as Clavien–Dindo Grade I or II.

Pneumothorax occurred in four cases (1.7%), with only one case requiring the placement of a pleural catheter (Grade III). Post-procedural hemoptysis or hemoptoic sputum was noted in 16 patients (6.7%); these events were predominantly Grade I, with 2 Grade II cases, and were managed conservatively with bed rest. No Grade IV (life-threatening) or Grade V (mortality) complications were observed. Exploratory univariate analysis showed an association between emphysema and Clavien–Dindo complication grade (*p* = 0.026), whereas no significant association were observed for COPD, pleural effusion, lymphadenopathy, smoking status, number of biopsy passes, needle size, or age. Given the limited number of complication events, analysis should be interpreted cautiously.

### 3.2. Assessing the Learning Curve

The diagnostic yield was identical between the first and second half of patients. The confirmation rate was 84.2% in both groups, with no statistically significant difference between the two periods (*p* = 1.00). Across procedural quartiles, the diagnostic yield was 85.0% in Q1, 83.3% in Q2, 90.0% in Q3, and 78.3% in Q4. Logistic regression analysis showed no significant association between case order and diagnostic yield (OR = 0.999; *p* = 0.64), indicating no evident learning-curve effect during the study period.

## 4. Discussion

Despite being performed during the early experience in US-TTCB of an interventional pulmonologist, US-TTCB achieved a diagnostic yield of 84.2% and a complication rate of 12.1% (only one Clavien–Dindo class III event) in our cohort of 240 patients. In the broad context of the medical literature, it is an acceptable rate of diagnosis and complications, and falls within the range reported. As a reference point, a meta-analysis of 12 studies (3830 procedures) reported for US-guided pulmonary/pleural biopsy a pooled sensitivity and specificity of 0.93 and 0.99, respectively, with an AUC of the summary receiver operator characteristic curve (SROC) of 0.95. Another aspect that was reported was a low overall complication rate of 3.6%, with only six pneumothoraces requiring chest tubes and one severe bleeding event [3].

When comparing our results to other studies, differences in diagnostic performance should be interpreted in the light of shared methodological challenges including the retrospective design, incomplete follow-up, variability in the definitions of non-diagnostic biopsy, or potential learning-curve effects during early implementation of the technique.

In this context, our diagnostic yield is comparable to that reported by Zhao et al., who observed a diagnostic accuracy of 82.0% from 501 US-TTCB [12].

Our result is also comparable with that of Guo et al., who reported a diagnostic accuracy of 81.8% in 648 ultrasound-guided procedures for 637 peripheral pulmonary lesions, with lesion size significantly affecting accuracy when measured both by ultrasound (*p* = 0.006) and CT (*p* = 0.001) [4].

Although higher diagnostic accuracy has been reported, direct comparisons require caution because of differences in case selection, study design, and outcome definitions. Li et al. reported 90.3% accuracy in 957 US-guided biopsies and all patients had more than 1 year of follow-up [13].

Sperandeo et al. reported 93.04% accuracy in 762 US-TTCB, but this was a prospective study with a highly selected cohort. Using predominantly a 16-G needle in a comparable cohort (263 patients; 150 men; mean age 60.7 ± 13 years), Ye et al. obtained adequate biopsy specimens in 92% of cases. Nevertheless, these comparisons should be interpreted cautiously because the endpoints differ [13,14,15].

From a pulmonologist–interventionist viewpoint, the diagnostic yield in our study closely matches the 83% success rate reported by Knox and Halligan in a small pulmonologist-performed series of 74 procedures. However, it is lower than the 94,6% adequate-sample rate reported by Murakmi et al., whose cohort included not only lung but also chest wall, pleural, and mediastinal lesions [16,17].

In addition, comparisons should account for differences in operator experience, needle gauge/biopsy technique, and lesion selection as these vary across studies. Operator experience differs across cohorts; for example, Murakami et al. report procedures performed by respiratory physicians under supervision of a faculty member with >15 years’ experience, which may stabilize performance and complication capture [17]. Similarly, Sperandeo et al. report procedures performed by highly experienced operators (>30 years) with repeat sampling in-session when macroscopic adequacy was uncertain, which may plausibly increase diagnostic performance compared with less standardized or less experienced settings [14].

Second, needle type and technique are not uniform. Murakami et al. used an 18 G core needle without a coaxial system, typically performing 2–3 passes, whereas our cohort used predominantly 16 G (with 18 G in a minority) and a freehand approach [17]. Notably, in Guo et al., procedures were performed using dedicated puncture probes with needle guide attachments (0°, 15°, and 30°), whereas our US-TTCB was performed freehand; these technical differences may limit direct comparability across studies [4].

Ye et al. specifically evaluated 16 G core needles and reported sample adequacy as the primary endpoint, emphasizing that gauge and endpoint choice affect comparability. Also, Li et al. included both 16 G and 18 G needles and identified 18 G as an independent risk factor for diagnostic failure, highlighting how needle selection can influence reported performance across cohort [13,15].

When it comes to lesion selection, it is worth noting that US-guided biopsy inherently favors pleural-contact lesions, but inclusion criteria and ‘target mix’ vary. Zhao et al. defined targets as lesions adjacent to or <3 cm from the pleura and excluded cases with uncertain diagnosis <6 months or lost to follow-up, which can increase apparent accuracy [12]. Murakami et al. included not only lung but also chest wall, mediastinal, and pleural lesions, and their conclusion emphasizes that safety and complication patterns depend on puncture site (intrapulmonary or extrapulmonary targets), limiting direct comparability with purely pulmonary cohorts [17].

Implementing a structured follow-up pathway for initially non-diagnostic biopsies was central to interpreting diagnostic performance in a clinically meaningful way. Non-diagnostic histology (e.g., non-specific inflammation, necrosis, scant cellularity, or inconclusive material) does not always represent true ‘failure’, as some cases subsequently declare their final diagnosis through longitudinal imaging, clinical evolution, or additional sampling. Therefore, follow-up was used to decide, wherever possible, if they were potentially misclassified as false negatives. However, a substantial proportion of initially non-diagnostic cases could not be reliably verified (25/52, 48%) due to loss to follow-up; we therefore retained these cases as non-diagnostic in the primary analysis, effectively accepting the initial pathology report and avoiding optimistic inflation of diagnostic yield through selective exclusion.

Using the Clavien–Dindo classification allows biopsy-related safety to be interpreted according to clinical severity rather than event frequency alone. In our study, 96.6% of events were Grade I–II and only one event was Grade III (0.4%), and it required a chest tube placement.

This crude result is similar to that of Guo et al., who reported complications in 12.8% of 648 US-guided procedures, including hemoptysis (8.0%), symptomatic pneumothorax (1.7%), and chest tube insertion (0.9%), although their study did not use Clavien–Dindo grading. It is also comparable to Knox and Halligan’s pulmonologist-performed series, where 4/33 transthoracic biopsies (12%) had complications, including two pneumothoraxes requiring chest tubes. Conversely, a lower complication rate was reported by Sperandeo et al. (0.79% for pneumothorax and 0.26% for hemoptysis) and Yamamoto et al. (3.3% in the US-guided group), likely reflecting differences in patient selection, lesion characteristics, operator experience, procedural technique, and complication definitions [4,6,14,16].

Importantly, Murakami et al. also used Clavien–Dindo grading and reported an overall complication rate of 7.1%, but all of them occurred after lung parenchymal biopsies, whereas the subgroup complication rate was 13.6%, closely approximating our rate; all pneumothoraxes were Grade I, and severe Grade IV–V events were limited to lung parenchymal targets. These findings support the interpretation that, although minor adverse events may be relatively frequent when systematically recorded, clinically significant morbidity after US-TTCB remains uncommon [17].

Exploratory analysis showed an association between emphysema and higher complication grade (*p* = 0.026), whereas COPD, pleural effusion, lymphadenopathy, smoking status, number of biopsy passes, needle size, and age were not significantly associated. Biologically, this is a plausible association as emphysematous lung parenchyma and bullous changes may increase vulnerability to air leak after pleural puncture. In a CT-TTLB, quantitative emphysema has been shown to predict post-biopsy pneumothorax, with Chami et al. reporting that percent emphysema was independently associated with pneumothorax after adjustment for various procedural-related variables. Similarly, in a US-guided 16 G biopsy series, Ye et al. found that bullae around the lesion independently predicted pneumothorax (OR 73.128; *p* = 0.026). However, not all studies confirm emphysema as an independent risk factor. Therefore, this association in our cohort should be interpreted cautiously because of the limited number of complication events, but it remains clinically relevant for pre-procedural risk stratification [7,15,18].

From a statistical standpoint, our findings fit well with other US-TT needle biopsy cohort models which demonstrated that lesion geometry/accessibility, rather than other variables, like patient history or demographics, are the strongest predictors of biopsy success. In a study by Jeon et al. (n = 97 procedures), lesion–pleural contact arc length was identified through multivariable logistic regression as the only independent predictor of a correct diagnosis (OR 1.16; 95% CI 1.04–1.30), and a threshold of >30 mm contact improved sensitivity and accuracy, highlighting how size and access to pleura tends to outperform other covariates statistically [19]. Regarding the multivariable modeling strategy, in many studies using large datasets, exploratory univariate analysis is first performed, and a parsimonious adjusted model is then constructed to reduce overfitting and unstable estimates, especially when categorical procedural variables are sparse. Li et al. (957 procedures) also emphasized the importance of handling indeterminate/non-diagnostic results rigorously and used multivariable logistic regression after univariate selection (*p* < 0.1), showing how analytic decisions affect diagnostic performance and which predictors are maintained after adjustment [13].

In our CT model, the multivariable analysis was restricted to the complete-case subset (n = 67) and included CT lesion size, COPD, and pleural effusion. In this context, the model should be interpreted as a secondary analysis rather than definitive predictive evidence. The model may also be underpowered and susceptible to selection bias and therefore should be interpreted cautiously. CT lesion size remained independently associated with diagnostic yield. However, given the findings of Jeon et al. and Lemieux et al., it also supports pleural contact length as a key determinant of sensitivity, rather than size alone. More recently, Lovato et al. used ROC analysis and found that depth, width, pleural contact, and number of biopsy passes had only fair discriminatory ability for obtaining an adequate biopsy (AUC 0.68–0.75), supporting the interpretation that single anatomic predictors are helpful for triage but are not definitive decision rules. In that context, our ROC result for CT lesion size (AUC 0.717) is best framed as an acceptable discrimination predictor, rather than an outlier finding. In addition, future models should include pleural contact length and lesion depth [19,20,21].

In our cohort, diagnostic yield was not improved in a time-dependent trend, which is plausible given the operator’s >10 years of experience in thoracic US, US-guided thoracocentesis, and bronchoscopy, skills that are strongly associated with US-TTCB (image optimization, needle visualization, and real-time hand–eye coordination). In the literature, ‘learning curves’ are described as being highly procedure-specific and often reflect a combination of baseline skill and system factors rather than case number alone. For example, in electromagnetic navigation bronchoscopy (ENB), cumulative sum control chart (CUSUM) learning curves among operators without prior ENB experience showed marked heterogeneity, with some operators requiring 25–30 cases to reach competency, while others had near competent performance from the outset, supporting the idea that transferable procedural-related skills can flatten early learning effects in some trainees [22].

Evidence from endobronchial ultrasound-guided transbronchial needle aspiration (EBUS-TBNA) learning-curve research reinforces two additional points: even ‘experienced bronchoscopists’ can have variable learning trajectories when adopting a new US-guided needle technique, and performance can be influenced by the learning environment and supervision. In a CUSUM chronological analysis of EBUS-TBNA training, all attending bronchoscopists had >5 years’ bronchoscopy experience but limited EBUS training at the start, and proficiency signals varied, with environmental support associated with improved performance. This aligns with the concept that our stable yield may reflect not only individual operator experience but also a mature, standardized workflow and case selection [23].

Finally, extrapolation from other US-guided biopsy domains supports the argument that prior procedural experience can shorten the path to proficiency. A recent CUSUM evaluation of US-guided pleural needle biopsy found proficiency after a relatively modest case volume and reported that operators with prior percutaneous biopsy experience reached an acceptable performance threshold sooner than those without such experience. Likewise, simulation-based training studies in renal and other US-guided biopsy settings reported improvement in trainee confidence and performance, supporting the idea that structured experience can reduce the visible learning curve [24,25,26]. Overall, our absence of an observable learning-curve effect is credible in the context of an operator with long-standing thoracic US and bronchoscopy experience, but we acknowledge that simple temporal splits can miss non-linear learning; future prospective work could add risk-adjusted CUSUM accounting for lesion complexity and process metrics (passes, procedure time, and repeat biopsy) to detect more subtle proficiency changes [23].

Key strengths of this study include STROBE-guided reporting, independent validation of extracted data, standardized complication grading using the Clavien–Dindo classification, and the inclusion of non-diagnostic cases in the diagnostic-yield denominator. Non-diagnostic biopsies were further evaluated using clinical, radiological, and, when available, histological follow-up, reducing the risk of yield underestimation. The single-operator design and consistent technical approach also enabled exploratory assessment of temporal performance.

This study has several limitations.

First, the retrospective design and reliance on hospital electronic medical records for follow-up may have introduced selection and information bias. Patients who sought care at other institutions or were lost to follow-up (25/52, 44%) could not be fully characterized, which may have limited reclassification of initially non-diagnostic results and potentially underestimated false-negative results.

Second, systematic confirmatory testing was not performed for initially diagnostic biopsies. Therefore, we cannot report diagnostic yield rather than comprehensive test-performance measures, like sensitivity and specificity, across the entire cohort.

Third, all procedures were performed in a single center by one highly experienced pulmonologist, which reduces technical variability and strengthens internal consistency (including the learning-curve assessment) but limits external validity. Ultrasound-guided and endoscopic biopsy techniques are known to be operator-dependent, and learning-curve analyses using CUSUM have demonstrated substantial between-operator variability, even among clinicians with prior procedural experience. Therefore, our diagnostic yield and safety profile may not directly translate into less experienced operators or centers with different workflows, case-misses, or training pathways [27].

In addition, complication evaluation depended on routine documentation, so minor or self-limited events may be under-reported compared with prospective surveillance, and we did not assess cost-effectiveness or patient-reported outcomes beyond routine peri-procedural comfort or pain documentation. These endpoints should be incorporated into future prospective, multicenter studies.

## 5. Conclusions

Pulmonologist-performed US-TTCB showed a high diagnostic yield and a low rate of major complications (Clavien–Dindo Grade III), supporting its role as an available bedside diagnostic procedure for selected pleural and peripheral pulmonary masses. Large CT dimension and COPD were associated with higher diagnostic success, while no significant learning-curve effect was observed. These findings should be interpreted in light of the retrospective, single-center, single-operator design and variable follow-up. Future prospective multicenter studies should validate CT lesion size thresholds, evaluate pleural contact length and lesion depth as predictors of diagnostic yield, and incorporate risk-adjusted CUSUM analyses with extended follow-up. Overall, our results suggest that US-guided biopsy is an effective diagnostic approach for pleural and peripheral lung masses, with a favorable safety profile.

## Figures and Tables

**Table 1:** Demographic and clinical characteristics.

Variable	Value
Patients, n	240
Age, years	67.0 (60.8–74.0)
Male sex	168 (70.0%)
Residence	121 rural (50.4%), 119 urban (49.6%)
Smoking status (n = 196)	76 current (38.8%), 87 former * (44.4%), 33 never (16.8%)
Pack-year index (n = 112)	40.0 (24.8–50.0)
COPD	107 (44.6%)
Pleural effusion	72 (30.0%)
Lymphadenopathy	67 (27.9%)
Emphysema	33 (13.8%)

* Prior cigarette use, no active smoking at biopsy, and ≥6 months of abstinence.

**Table 2:** Lesion characteristics.

Variable	Value
Lesion location (*n* = 239)	107 upper lobe (44.8%), 6 middle lobe (2.5%), 125 lower lobe (52.3%), 1 other (0.4%)
CT lesion size **, mm (*n* = 67)	60.0 (43.5–87.5)
Ultrasound lesion size, mm	40.0 (30.0–60.0)
Malignant lesions	181/240 (75.4%)

** The largest diameter of the lesion on a CT image in a lung window setting.

**Table 3:** Procedural and technical characteristics.

Variable	Value
Number of biopsy passes	2 (2–3)
Needle size	208/240 16 G (86.7%), 32/240 18 G (13.3%)
Patients position during biopsy (n = 240)	69 dorsal decubitus (28.8%), 67 ventral decubitus (27.9%), 39 sitting (16.2%), 35 left lateral decubitus (14.6%), 30 right lateral decubitus (12.5%)

**Table 4:** Primary endpoints.

Variable	Value
Diagnostic yield	202/240 (84.2%)
Any complication	29/240 (12.1%)
Major complication (Clavien–Dindo Grade III)	1/240 (0.4%)

**Table 5:** Histologic types identified through US-TTCB and ultrasound lesion size in mm, with median and IQR.

Histologic Type	*N* (%)	US Lesion Size, mm (Median, IQR)
Adenocarcinoma	85 (35.4)	45 (30–60)
Squamous cell carcinoma	37 (15.4)	50 (38–80)
Inflammatory lesion	30 (12.5)	30 (30–44.5)
Metastatic lesions from extrapulmonary primary tumors	21 (8.8)	35 (25–40)
Necrosis	17 (7.1)	40 (30–40)
Undifferentiated carcinoma	12 (5.0)	50 (38.8.5–62.5)
Small-cell carcinoma	10 (4.2)	49 (34–60)
Large-cell neuroendocrine carcinoma	7 (2.9)	50 (47.5–64)
Adenosquamous carcinoma	3 (1.2)	26 (25.5–33)
Pleural inflammation	3	25 (22.5–37.5)
Solitary fibrous tumor	3	80 (70–105)
Lipoma	2 (0.8)	47.5 (38.8–56.3)
Lymphoma	2	52.5 (48.8–56.3)
Pleural mesothelioma	2	35 (27.5–42.5)
Capillary hemangioma	1 (0.4)	40
Tuberculoid granuloma	1	30
Plasmacytoma	1	45
Blastoma	1	70
Reduced cellularity	1	50
Inconclusive	1	80

**Table 6:** Exploratory univariate analysis.

Variable	Diagnostic Yield (*n* = 202)	Non-Diagnostic Biopsy (*n* = 38)	*p*-Value
Age, years	67.0 (60.2–74.0)	67.0 (61.2–73.8)	0.8305
Sex, male	140 (69.3%)	28 (73.7%)	0.7284
Smoking status			0.17
Current smoker	66 (40.2%)	10 (31.2%)	
Former smoker	74 (45.1%)	13 (40.6%)	
Never smoked	24 (14.6%)	9 (28.1%)	
Pack-years index	40.0 (25.0–50.0)	30.0 (20.0–50.0)	0.2286
Secondary determinations	41 (20.3%)	10 (26.3%)	0.29
Emphysema	64 (31.7%)	11 (28.9%)	0.84
COPD	59 (29.2%)	5 (13.2%)	0.068
Pleural effusion	33 (16.3%)	11 (28.9%)	0.089
Lymphadenopathy	54 (26.7%)	8 (21.1%)	0.46
Number of biopsy passes	2 (1–2)	1 (1–2)	0.38
Needle size, gauge	18 (18–20)	19 (18–20)	0.65
Lesion location			0.3
Upper lobe	97 (48.0%)	15 (39.5%)	
Middle lobe/lingula	21 (10.4%)	6 (15.8%)	
Lower lobe	84 (41.6%)	17 (44.7%)	
Lesion size on CT (mm)	69.6 ± 35.4	44.0 ± 25.1	0.0098
Lesion size on ultrasound (mm)	45.0 (30.0–60.0)	37.5 (30.0–49.0)	0.0402
Patient position during biopsy			0.045
Dorsal	63 (31.2%)	6 (15.8%)	
Right lateral decubitus	25 (12.4%)	5 (13.2%)	
Left lateral decubitus	30 (14.9%)	5 (13.2%)	
Sitting	35 (17.3%)	4 (10.5%)	
Ventral	49 (24.3%)	18 (47.4%)	

**Table 7:** Multivariable logistic regression analysis of stable and interpretable factors associated with diagnostic yield. Model based on 67 complete cases.

Variable	Adjusted OR	95% CI	*p*
Lesion size on CT	1.03	1.00–1.05	0.022
COPD	2.3	1.06–4.96	0.034
Pleural effusion	0.5	0.24–1.04	0.063

## Data Availability

The data supporting this study are available from the authors upon reasonable request. They are not publicly available because some variables could allow indirect participant identification.

## Supplementary Materials

The following supporting information can be downloaded at: https://www.mdpi.com/article/10.3390/diagnostics16121913/s1, Supplementary File S1.

## Appendix A

**Table A1:** Definition and grading of Clavien–Dindo classification adapted for US-TTCB, based on the highest level of therapy required. Major complications: Clavien–Dindo grade ≥III.

Clavien–Dindo Grade	Definition (Therapy-Based)	Examples Applicable to US-TTCB
I	Deviation from normal course without pharmacological treatment or invasive interventions (allowed: antiemetics, antipyretics, analgesics, and electrolytes)	Transient vasovagal episode resolved with observation; mild pain controlled with simple analgesics; small asymptomatic pneumothorax managed by observation
II	Requires pharmacological treatment beyond Grade I (e.g., antibiotics, blood transfusion, and specific drug therapy)	Hemoptysis treated with medication; suspected infection treated with antibiotics; bleeding requiring transfusion
III	Requires radiologic/endoscopic/surgical intervention with or without general anesthesia	Pneumothorax requiring needle aspiration or chest tube drainage; image-guided hemostatic intervention or surgical control of bleeding
IV	Life-threatening complications	Respiratory or multi-organ failure requiring intensive care unit management
V	Death	Procedure-related death
